# Microstructure Evolution and Mechanical Properties of AlCoCrFeNi_2.1_ Eutectic High-Entropy Alloys Processed by High-Pressure Torsion

**DOI:** 10.3390/ma17122954

**Published:** 2024-06-17

**Authors:** Fanghui Wang, Chaogang Ding, Zhiqin Yang, Hao Zhang, Ziheng Ding, Hushan Li, Jie Xu, Debin Shan, Bin Guo

**Affiliations:** 1 Key Laboratory of Micro-Systems and Micro-Structures Manufacturing of Ministry of Education, Harbin Institute of Technology, Harbin 150001, China; 2 National Key Laboratory for Precision Hot Processing of Metals, Harbin Institute of Technology, Harbin 150001, China; 3 School of Materials Science and Engineering, Harbin Institute of Technology, Harbin 150001, China; 4 Department of Materials Science and Engineering, Pohang University of Science and Technology, Pohang 37673, Republic of Korea

**Keywords:** AlCoCrFeNi_2.1_ eutectic high-entropy alloys, high-pressure torsion, microstructure evolution, mechanical properties

## Abstract

High-entropy alloys (HEAs) have garnered significant attention for their exceptional properties, with eutectic high-entropy alloys (EHEAs) emerging as particularly notable due to their incorporation of eutectic structures comprising soft and hard phases. This study investigated the influence of shear strain on the microstructural refinement and mechanical properties of AlCoCrFeNi_2.1_ EHEAs, which were subjected to high-pressure torsion (HPT) at room temperature under a pressure of 6 GPa across 0.5 to 3 turns, compared to the initial material. After HPT treatment, significant grain refinement occurred due to strong shear strain, evidenced by the absence of B2 phase peaks in X-ray diffraction (XRD) analysis. Microhardness increased substantially post-HPT, reaching a saturation point at approximately 575 HV after three turns, significantly higher than that of the original sample. Moreover, the ultimate tensile strength of HPT-treated specimens reached around 1900 MPa after three revolutions, compared to approximately 1100 MPa for the as-cast alloy, with a mixed fracture mode maintained. This investigation underscores the efficacy of HPT in enhancing the mechanical properties of AlCoCrFeNi_2.1_ EHEAs through microstructural refinement induced by shear deformation, offering insights into the design and optimization of advanced HEAs for various engineering applications.

## 1. Introduction

High-entropy alloys (HEAs) have garnered significant attention due to their outstanding properties [1,2,3]. Among the plethora of developed HEAs, eutectic high-entropy alloys (EHEAs) have emerged as particularly noteworthy owing to the incorporation of eutectic structures comprising soft and hard phases [4,5]. In 2014, EHEAs with a nominal composition of AlCoCrFeNi_2.1_ were proposed by Lu et al. [4]. Based on the research conducted by Lu et al. and Wani et al. [4,6], it has been determined that the AlCoCrFeNi_2.1_ EHEAs contains a hard-body-centered cubic (BCC) phase and a soft-face-centered cubic (FCC) phase. Additionally, due to the ordered nature of these phases, the BCC phase and FCC phase were defined as the B2 phase and L1_2_ phase, respectively [4,6,7]. Due to the advantages of AlCoCrFeNi_2.1_ EHEAs, including lightweight, high strength, corrosion resistance, excellent manufacturability, and superior impact and creep resistance, particularly under extreme conditions such as high and low temperatures, it is anticipated that this material will be utilized in the manufacturing of large, complex components such as propellers [8,9,10,11]. Currently, it is gaining considerable recognition in the aerospace, automotive, and various other industries [9,12].

To delve deeper into the performance of AlCoCrFeNi_2.1_ EHEAs, numerous scholars have conducted extensive research on their microstructure and mechanical properties. Asoushe et al. [13] investigated the microstructure evolution of AlCoCrFeNi_2.1_ EHEAs during thermomechanical processing across temperatures ranging from 25 to 500 °C. They observed insignificant changes in the internal microstructure at higher temperatures, resulting in inconspicuous alterations in the mechanical properties of the alloys, thus confirming the thermomechanical stability within this temperature range. Wani et al. cold-rolled AlCoCrFeNi_2.1_ EHEAs and subsequently annealed them at 800 °C, resulting in the destruction of the internal layer structure and the formation of ultrafine equiaxed grains to enhance the strength [14]. Zheng et al. prepared AlCoCrFeNi_2.1_ EHEAs using directional solidification technology and explored their mechanical properties [15]. They found that at a withdrawal rate of 5 μm/s, the microstructure comprised the primary FCC phase and the eutectic lamellar BCC phase, with a fracture strength of approximately 700 MPa. Furthermore, an increase in the withdrawal rate led to higher fracture strength but a gradual decrease in elongation. Using conventional manufacturing techniques increases strength, but is not enough to meet actual manufacturing requirements. Huang et al. [5] utilized laser metal deposition (LMD) to create a refined uniform structure, significantly enhancing the tensile strength and elongation of AlCoCrFeNi_2.1_ EHEAs due to the refinement of the internal microstructure, which improved strain hardening and dislocation accumulation capabilities. Chen et al. [16] embedded the hard-layered B2 phase within the FCC phase matrix of AlCoCrFeNi_2.1_ EHEAs using an innovative process involving multi-stage cold drawing and subsequent heat treatment. Meanwhile, Chen et al. [17] fabricated nano lamellar-structured AlCoCrFeNi_2.1_ EHEAs via selective laser melting (SLM), resulting in improved mechanical properties [16,17]. Wang et al. conducted in situ tensile tests on AlCoCrFeNi_2.1_ EHEAs, characterized the microstructure through transmission electron microscopy (TEM), and discovered that cross-slip and dislocation substructure in the FCC phase contributed to strain hardening, while the BCC phase enhanced the strength through precipitation strengthening [18]. The findings of Wang et al. indicated that heterogeneous structures could resolve the inverse relationship between strength and ductility by employing multiple strengthening mechanisms. Regulating the balance between microstructure and crystal defects within AlCoCrFeNi_2.1_ EHEAs by existing techniques as a way to improve mechanical properties plays an important role in meeting the needs of industrial development. Recently, the AlCoCrFeNi_2.1_ EHEAs has been studied by many scholars using heat treatment processes, drawing processes, and additive manufacturing. However, its complex processing requirements and suboptimal mechanical properties have made it challenging to meet the demands of advanced industrial production.

Grain refinement represents a theoretically effective approach to augmenting increased metal strength while preserving elongation [19], with the high-pressure torsion (HPT) process standing as a quintessential method of severe plastic deformation (SPD) for fabricating disc-like nanostructured materials, leveraging substantial shear strain and high hydrostatic pressure often reaching several GPa [20,21]. Edalati et al. [22] investigated the hardness of dual-phase AlCrFeCoNiNb alloy post-HPT, which escalated to 1030 HV, attributed to pronounced lattice distortion engendered by nanoparticle formation averaging 10 nm in size, alongside the proliferation of dislocations and solid solutions. Zheng et al. subjected iso-atomic FeNiCoCu HEAs and Ti and Al added (FeNiCoCu)_86_Ti_7_Al_7_ HEAs to HPT spanning up to 10 revolutions, revealing a resultant dual-phase nanocrystalline structure post-HPT, with grain boundary strengthening and dislocation strengthening serving as primary reinforcing mechanisms [23]. Additionally, Liu et al. administered a single-turn HPT treatment to AlCrFe_2_Ni_2_ HEAs, inducing transformation from a biphasic structure comprising BCC and FCC phases to a singular FCC phase structure, with ensuing room temperature tensile tests indicating heightened hardness and strength but diminished elongation (about 33%) [24]. Duan et al. showcased MoNbTaTiVZr dual-phase HEAs via HPT up to 40 turns, noting enhanced microhardness and more uniform elemental distribution [25]. Through dissecting the mechanism behind microhardness augmentation under varying strains, it was hypothesized that lower strain-induced hardness escalation primarily stemmed from heightened dislocation density and grain refinement, while a sharp rise in hardness under higher strains was predominantly attributed to ultra-fine crystal microstructure formation. Therefore, the severe deformation induced by the HPT process can be utilized to refine the internal grains, thereby enhancing the strength of the alloy to meet the production requirements for high-strength materials.

In this study, the influence of applied shear strain on microstructure evolution and mechanical properties of AlCoCrFeNi_2.1_ EHEAs was scrutinized via HPT at distinct revolution intervals. The uneven distribution of shear force induced by the HPT process results in varying levels of refinement within the internal microstructure, thereby producing a gradient distribution of microstructural features. Microstructural examination and tensile testing unveiled substantial enhancement in strength and microstructural refinement facilitated by significant shear deformation induced by HPT. Furthermore, the mixed fracture mode was maintained after the HPT process compared to the as-cast alloy. The high-strength AlCoCrFeNi_2.1_ EHEAs produced through the HPT process provide both a theoretical foundation and a practical basis for the extensive engineering application of the AlCoCrFeNi_2.1_ EHEAs.

## 2. Materials and Methods

The AlCoCrFeNi_2.1_ EHEAs ingot, comprising elements in molar ratios, was fabricated via arc melting, utilizing high-purity metals (more than 99.99%) in an argon atmosphere. The chemical composition of the AlCoCrFeNi_2.1_ EHEAs is detailed in Table 1. To ensure compositional uniformity, the ingot, weighing approximately 2.5 kg, underwent five successive re-meltings. Subsequently, disk-shaped specimens, measuring 10 mm in diameter and approximately 1.0 mm in thickness, were machined from the ingot and subjected to HPT treatment. Prior to HPT processing, each specimen underwent meticulous polishing on both sides, achieving a final thickness of 1.5 mm. The specimens were then positioned in the recess on the surface of the lower anvil, with the two anvils subsequently brought together to apply pressure, with further processing details given in prior publications [26,27,28,29]. HPT was conducted at room temperature under a pressure of 6 GPa, with varying numbers of revolutions denoted as *N*, including 0.5, 1, and 3 turns, at a rotation speed of 1 rpm. Process parameters were selected to promote a more uniform refinement of the microstructure [26,27,28,29]. For each process condition, five samples were prepared to ensure the precision of the subsequent experimental data.

**Figure 1:**
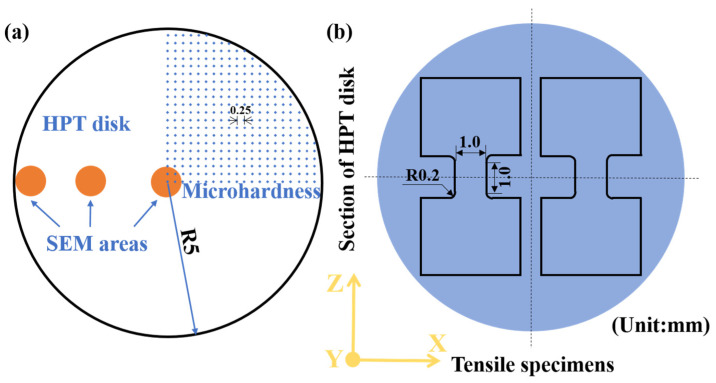
(**a**) Schematic illustration of the positions for microhardness measurements and SEM observation of the disk after HPT, (**b**) the dimensions and positions of the micro tensile samples in HPT disk.

In order to accurately measure microhardness, the disk-shaped samples were grounded and polished using sandpapers to attain mirror-like surfaces. Symmetrical shear deformation was applied to the entire HPT-treated samples, resulting in a radially symmetric distribution of microhardness. The 1/4 area of the disk surface was designated as the microhardness measurement zone, with a 0.25 mm interval between each microhardness point, as depicted in Figure 1a. Microhardness testing was conducted using a semi-automatic Vickers hardness tester with an applied force of 200 g and a dwell time of 10 s, traversing from the center to the edge. Microhardness measurements were conducted on five samples, and the resultant average value was utilized as the microhardness value at each specific point. Subsequently, dog-bone-shaped tensile specimens were extracted from both the HPT-treated and original samples, featuring specific dimensions as illustrated in Figure 1b. Mechanical property evaluations were carried out at room temperature with a strain rate of 10^−3^ s^−1^ by a universal testing machine (Instron 5530, Norwood, MA, USA, 50 kN). On account of the size of the tensile samples post-HPT, digital image correlation (DIC) was employed to accurately determine strain corresponding to stress. It is noteworthy that prior to tensile testing, the specimens were polished with 2000# sandpaper to mitigate the influence of surface roughness on mechanical properties. Subsequently, the polished surface was coated with a matte white primer and toner to create surface spots, which facilitated post-processing analysis by the DIC technique. Each tensile experiment was replicated three times to mitigate experimental variability, thereby enhancing the scientific validity and precision of the experiments.

Initial and HPT-treated samples were examined via X-ray diffraction (XRD) employing Cu Kα radiation, with a wavelength of 1.5405 Å, to elucidate phase structures. The detected angle range was selected from 20° to 100°, with a scanning step of 0.05° and a scanning speed of 4° per minute. Then, the Scherrer equation was used to calculate the grain size by Jade 6.5 software [30]. Additionally, a scanning electron microscope (SEM, ZEISS Gemini 560, Oberkochen, Germany) equipped with a field-emission gun was utilized at an accelerating voltage of 30 kV to characterize the microstructure and second-phase evolution. Chemical element distribution was further analyzed via energy-dispersive X-ray spectroscopy (EDS). As illustrated in Figure 1a, three circular areas with a diameter of 1 mm were selected to observe the microstructure evolution.

## 3. Results

### 3.1. Microstructure Evolution and Phase Composition

**Figure 2:**
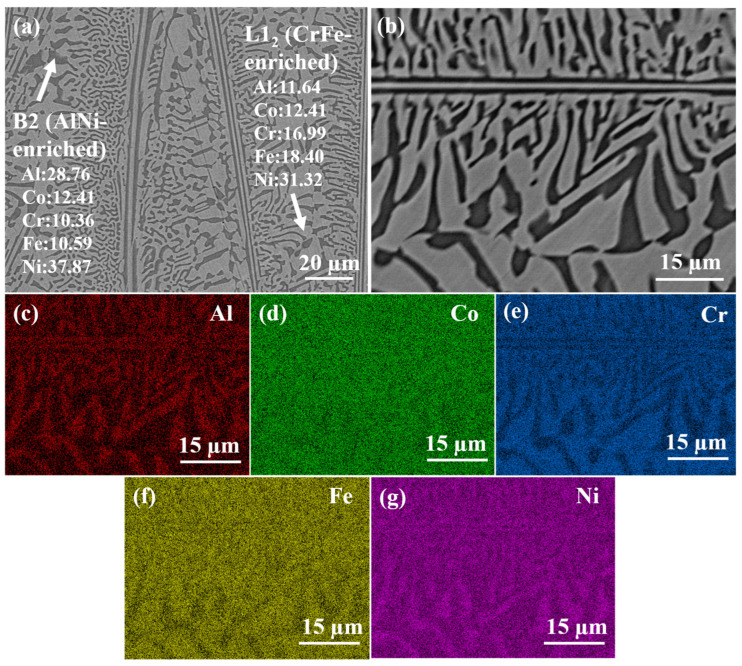
(**a**,**b**) SEM image of the as-cast AlCoCrFeNi_2.1_ EHEAs, and SEM-EDS elemental mapping of (**c**) Al, (**d**) Co, (**e**) Cr, (**f**) Fe and (**g**) Ni.

To gain deeper insights into the microstructure of the as-cast AlCoCrFeNi_2.1_ EHEAs, it is exhibited in Figure 2 [31]. The average grain size of the as-cast AlCoCrFeNi_2.1_ EHEAs is approximately 86 ± 5 µm. Figure 2a,b illustrate the eutectic structure of the AlCoCrFeNi_2.1_ EHEAs, while Figure 2c–g depict the distribution of elements corresponding to Figure 2b. Moreover, the heterogeneity of chemical elements detected by SEM-EDS analysis was demonstrated in Figure 2c–g. The results revealed that the black phase corresponds to Al and Ni-enriched regions, whereas the off-white phase corresponds to Cr and Fe-enriched regions [4,6]. The specific chemical elements are delineated in Figure 2a.

**Figure 3:**
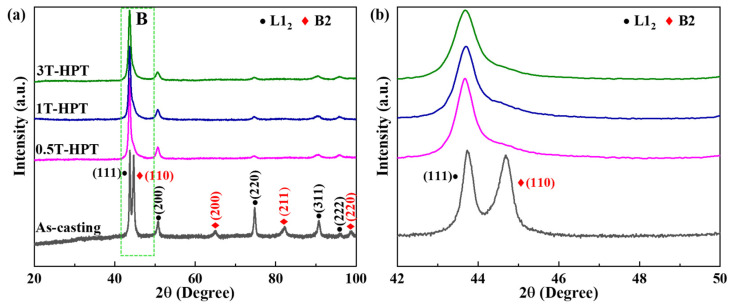
(**a**) XRD analysis for the sample with as-cast and after HPT through 0.5, 1 and 3 turns, (**b**) enlarged images of region B in (**a**).

**Figure 4:**
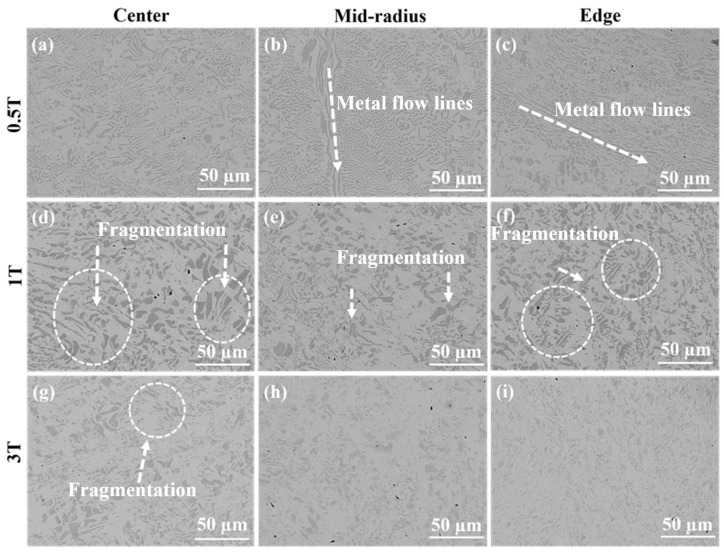
The microstructure after HPT for 0.5 turns (**a**–**c**), and 1 turn (**d**–**f**) and 3 turns (**g**–**i**) at the center, mid-radius and edge.

Figure 3a illustrates the XRD patterns of AlCoCrFeNi_2.1_ EHEAs in their as-cast state and following various numbers of HPT turns, while selected patterns within the 42° to 50° range are clearly presented in Figure 3b. As the XRD detection was conducted on the polished disk surface, the average level of varying shear strain in different regions along the radius direction for different HPT processes could be represented by the intensity of the diffraction peak of different phases, which was highly agreed with the previous research [24]. The average grain sizes of the samples were determined using XRD data analysis, revealing grain sizes of 171 nm, 161 nm, and 142 nm after undergoing HPT for 0.5 turns, 1 turn, and 3 turns, respectively, which was similar to the previous study [29]. However, as seen in Figure 3b, the diffraction peaks of HPT-treated samples were wider than those of the initial as-cast sample due to lattice strain induced by dislocation formation and grain size reduction [22,24]. Furthermore, the XRD pattern of HPT-treated samples exhibited the weakened intensity of the L1_2_ phase in comparison to the peaks of the original alloy, indicating gradual refinement of the L1_2_ phase through HPT treatment, as reported by Yang et al. [32]. Therefore, the widening and weakening of diffraction peaks are attributed to grain refinement induced by shear deformation during HPT treatment. As depicted in Figure 3b, a very small peak corresponding to the B2 phase was observed at approximately 43° in the samples by HPT. It is noteworthy that the diffraction peak of the B2 phase was nearly absent after HPT, consistent with prior findings [33,34,35,36]. It has been suggested in previous reports that this observation could be attributed to the shear strain induced during the HPT deformation process, resulting in the splitting, fragmentation, and reduction in grain size of the second phase [33]. Additionally, it may also be due to grain boundary slip, causing the small grains of the second phase to fracture, leading to thin layers on the grain boundaries of the primary phase [36]. Consequently, characteristic peaks of the second phase are undetectable once grain size reaches a certain threshold. This observation aligns with the results presented in Figure 4, indicating that samples subjected to the HPT process remain dual-phase alloy.

Figure 4 presents the microstructure of samples subjected to HPT from 0.5 to 3 turns. Distinctly, the different microstructure was observed under different HPT treatments and with different regions of the same HPT sample. The original microstructure was still retained at the center of the sample treated with 0.5 turns by HPT in Figure 4a. Moreover, the metal flow lines were explicitly observed at the mid-radius and edge regions, with the L1_2_/B2 phases in these regions becoming kinked and bent along the metal flow lines after 0.5 turns of HPT, as depicted in Figure 4b,c. However, as the number of HPT turns increased, the microstructure at the mid-radius and edge regions became more homogeneous, and the metal flow lines disappeared. Consequently, due to the prominent effect of shear force introduced through HPT, the fragmentation and refinement of microstructure were visibly found (as shown in the markings of Figure 4). Nevertheless, the microstructure at the center was less refined than that of other regions due to the lower shear strain, as shown in Figure 4d–i. These mentioned results confirmed that the level of shear deformation by HPT farther from the center was higher than that near the center. In contrast, when the HPT revolution reached three turns, the microstructure was performed considerably more uniformly owing to the saturation of shear deformation. On the whole, the eutectic microstructure observed in the initial alloy was not presented in the HPT samples due to the severe shear deformation [37], which destroyed the original microstructure, as displayed in Figure 4. In general, the level of the microstructure refinement was directly proportional to the number of HPT revolutions and the distance from the center, with the microstructure evolution of HPT samples strongly dependent on the position and the revolutions of HPT [24]. Therefore, the variations at the edge were notably greater than those at the center. However, according to earlier studies [32,37], when the shear deformation caused by the HPT process reached a certain level, the effect of grain refinement became less pronounced, as presented in Figure 4h,i. The results indicate that the HPT process could effectively facilitate the refinement of the AlCoCrFeNi_2.1_ EHEAs’ microstructure and disrupt the internal phase composition, particularly the B2 phase. Furthermore, the B2 phase gradually transforms into small particles with the increase in shear deformation induced by the HPT process, thereby explaining the disappearance of the B2 phase peak following HPT. Additionally, as illustrated in Figure 3, the peaks corresponding to the L1_2_ phase gradually diminish, suggesting that the L1_2_ phase has also undergone refinement after HPT.

### 3.2. Microhardness Evolution

**Figure 5:**
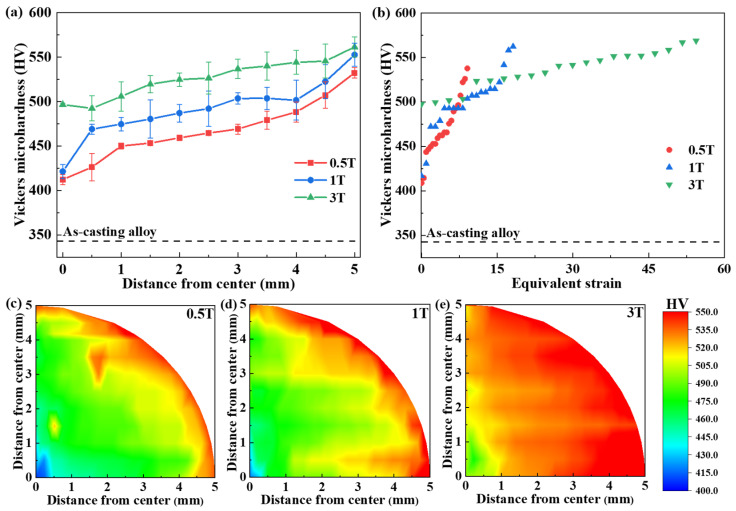
The Vickers microhardness curves with (**a**) distance from the center and (**b**) equivalent strain of HPT samples through 0.5, 1 and 3 turns, and color-coded contour mapping for (**c**) 0.5, (**d**) 1 and (**e**) 3 turns.

Figure 5a illustrates the correlation between the microhardness and distance from the center following HPT with varying revolutions. The microhardness of the as-cast AlCoCrFeNi_2.1_ EHEAs was approximately 343 HV, as denoted by the dashed line in Figure 5a. As depicted in Figure 4, under the same number of torsional turns, the microstructure of the edge region exhibits greater refinement compared to that of the central region, owing to the uneven distribution of shear force introduced by the HPT process. Furthermore, with an increase in the number of torsional turns, the microstructure of samples subjected to higher turns became finer than that of samples subjected to fewer turns. Consequently, the level of microstructure refinement was higher in the edge region than in the central region, and the microstructure refinement level of samples with a higher number of torsional turns surpasses that of samples with fewer torsional turns. It is worth noting that microhardness increases with increasing microstructure refinement within a certain threshold [29]. Due to the uneven refinement of microstructure induced by shear deformation during HPT, the microhardness in the central region was significantly lower than that at the edge. This observation was consistent with the findings presented in the contour maps in Figure 5c–e. As shown in Figure 5c–e, the microhardness of the central region gradually increased with an increase in the number of turns, and according to previous reports, that was due to the heightened strain gradient introduced by HPT, leading to the generation of geometrically necessary dislocations (GNDs) [29,38]. With the number of revolutions reaching a certain level, the high microhardness zone progressively moved to the center until the microhardness reached saturation. Thus, the microhardness increased with both the distance from the center and the number of HPT revolutions. According to previous work [39], the microhardness evolution of AlCoCrFeNi_2.1_ EHEAs followed the pattern of hardening without recovery, meaning that the microhardness gradually increased with increasing shear deformation.

According to Figueiredo et al. [40], the amount of rotation applied to the sample determined the equivalent strain. During HPT, lower strain levels were observed near the center, while the strain substantially increased away from the center. It was demonstrated that the distribution of equivalent strain during the HPT process is non-uniform. As illustrated in Figure 4, microhardness in the HPT process was related to the level of microstructure refinement, which in turn was related to the equivalent strain. Thus, microhardness increased with increasing equivalent strain below a certain threshold [40]. By calculating the equivalent strain *ε_eq_* using Equation (1) [40], the relationship between equivalent strain and microhardness was examined:

(1) $$\varepsilon_{eq}=\frac{2\pi rN}{h\sqrt{3}},$$

where *r* is the radial distance from the center of the disk, *N* is the number of torsion revolutions and *h* is the thickness of the disk.

The variations in microhardness with equivalent strain at different HPT revolutions are presented in Figure 5b. With the increase in equivalent strain, two saturation stages were observed during the process of microhardness enhancement, which was in line with the report of Duan et al. [25]. It has been suggested by Edalati et al. [22] that the saturation state arose from the equilibrium between defect generation and annihilation through dynamic recovery, dynamic recrystallization, and grain boundary migration. As displayed in Figure 5d,e, the microhardness rapidly increased to 475 HV when the equivalent strain was low. attributed to the required shear strain for shear deformation during HPT, as reported [25]. The microhardness reached the first saturation state at an equivalent strain of approximately 10 before gradually rising to 500 HV. Subsequently, the microhardness steadily increased to 550 HV, reaching a second saturation point at an equivalent strain of around 40, which was consistent with the studies of many scholars [22,24,25,33,37,41,42,43].

Therefore, the aforementioned results indicated that the microhardness was significantly improved during HPT compared to the as-cast sample, owing to the high density of dislocations and grain refinement induced by HPT. Once the shear deformation reached the threshold value, the microhardness tended toward a saturated state due to the equilibrium between defect generation and annihilation.

### 3.3. Tensile Properties

**Figure 6:**
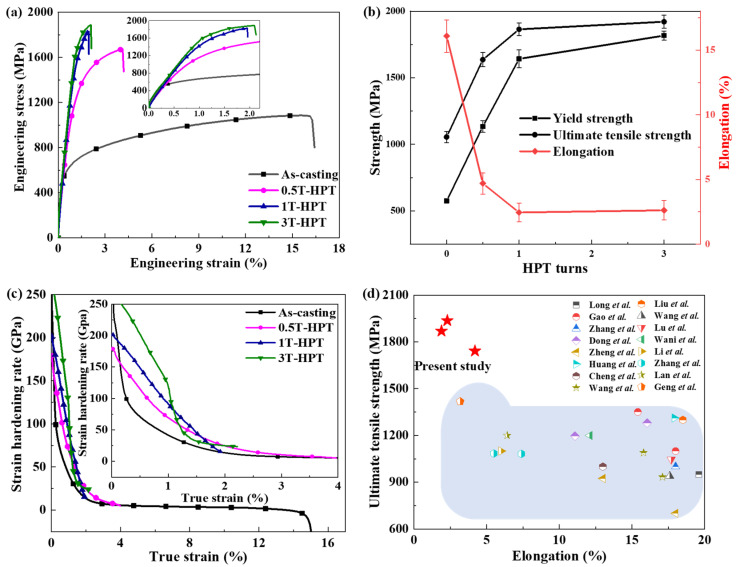
Tensile properties in both the as-cast condition and after HPT with various turns: (**a**) engineering stress-engineering strain curves, (**b**) UTS, YS and elongation derived from the engineering stress-engineering strain curves, (**c**) strain hardening rate curves with true strain and (**d**) contrast graph between UTS and elongation in this study and previously reported [5,8,14,15,18,31,56,57,58,59,60,61,62,63,64,65].

The engineering stress and engineering strain curves of AlCoCrFeNi_2.1_ EHEAs in both as-cast and HPT conditions are depicted in Figure 6a with an initial strain rate of 1 × 10^−3^ s^−1^. Figure 6b presents the ultimate tensile strength (UTS) and yield strength (YS, at 0.2% offset) versus the number of HPT turns. The significantly enhanced values of YS and UTS were observed after HPT, albeit with a considerable reduction in total elongation compared to the as-cast alloy. The HPT-treated alloys exhibited an elongation of approximately 2% following 1 and 3 turns, whereas the as-cast counterpart displayed an elongation of around 15%. The phenomenon, characterized by an increase in strength but a decrease in elongation after HPT, has been consistently observed in numerous reports [24,44,45,46,47,48,49]. The elongation of AlCrFe_2_Ni_2_ subjected to HPT decreased from 38% to 5% [24]. The medium entropy alloy CoCrNi was subjected to HPT by Schuh et al. [47], resulting in a decrease in elongation from 37% to 5%. Moreover, the V_10_Cr_15_Mn_5_Fe_35_Co_10_Ni_25_ after HPT was studied, and the reduction in elongation was approximately from 48% to 6% [48,49]. Similar observations have been reported in Al [44], Ni [45] and Cu alloys [46]. The higher YS and UTS values were attributed to the refinement of microstructure and the generation of a high dislocation density through HPT. However, the limited ductility observed in the HPT samples was attributed to the emergence of microscopic cracks caused by the high density of dislocations and the reduction in grain size [24,50,51,52]. Additionally, an intriguing observation was made in Figure 6a,b, where the elongation of samples subjected to three turns of HPT was higher than that of samples subjected to one turn, which was similar to the results of Kim et al. for Al7075 alloy [53]. The anomalous increase in ductility was attributed to the fragmentation and dissolution of brittle phases, with the improvement in ductility in the three-revolution sample after HPT attributed to the refinement of the B2 phase by severe shear deformation during HPT treatment, as shown in Figure 4.

In Figure 6c, the strain hardening rate of both as-cast and HPT alloys during the tensile test are presented. Here, the strain hardening rate (θ = dσ/dε) was derived from the corresponding true stress-true strain curve and plotted as a function of the true strain. During the elastic to plastic transition stage, the strain hardening rate of as-cast specimen rapidly decreased, followed by a monotonic decrease with increasing true strain, and a rapid decrease at the late deformation stage due to necking. According to the study [24], the decreasing strain hardening rate of as-cast counterpart was attributed to dislocation slip during tensile deformation. However, with the exception of the alloy subjected to 0.5 turns of HPT, whose strain hardening rate was similar to that of original as-cast specimen, the strain hardening rate of alloys after other revolutions rapidly decreased due to the higher dislocation density introduced during HPT [50,51,52]. As illustrated in Figure 6d, a significant improvement in strength was exhibited during HPT compared to heat treatment following rolling. Compared to other conventional forming processes, the intense shear deformation introduced by HPT refined the internal microstructure, leading to an increase in strength as the microstructure became thinner, as shown in Figure 6d. This concept offered insights into the preparation of high-strength alloys. However, the ductility of HPT alloys was weakened. In a word, the HPT resulted in an imbalance between strength and ductility. Therefore, in order to address this imbalance phenomenon, it was believed that heat treatment could improve ductility at the expense of some strength, as previously reported [54,55]. However, for AlCoCrFeNi_2.1_ EHEAs, this requires further experimental verification.

### 3.4. Fracture Morphology

**Figure 7:**
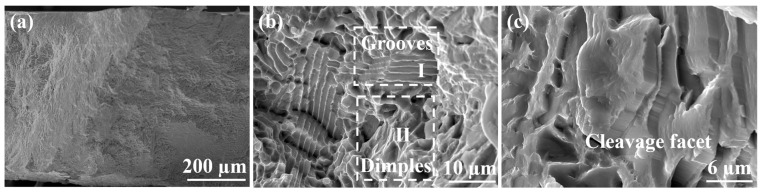
Tensile fracture surface of as-cast AlCoCrFeNi_2.1_ EHEAs: (**a**) macroscopic fracture morphology, (**b**) mixed fracture morphology and (**c**) cleavage facet morphology.

The tensile fracture surface morphologies of as-cast AlCoCrFeNi_2.1_ EHEAs were described to reveal the fracture mechanism, as depicted in Figure 7. Figure 7a shows the uneven macroscopic fracture morphology. There were two diverse fracture patterns, among which one was the ductile fracture mode by the L1_2_ phase and the other was the brittle transgranular fracture mode by the B2 phase, as illustrated in earlier reports [5,66]. Additionally, grooves (marked I) and dimples (marked II) were discovered in Figure 7b, which was consistent with the report [67]. According to the study of Tang et al. [68], this phenomenon was caused by the deviation of the lamellar eutectic structure orientation from the direction of tensile stress. When they were parallel to each other, the brittle fracture mode formed the bottom of the grooves, while the edge of the grooves was formed by ductile fracture mode [8,69], as shown in Figure 7b. Furthermore, the presence of cleavage facets, a typical brittle fracture mode, was found, as presented in Figure 7c. Consequently, the tensile fracture mode of as-cast AlCoCrFENI_2.1_ EHEAs was characterized as a mixed fracture composed of ductile fracture and brittle fracture [8].

**Figure 8:**
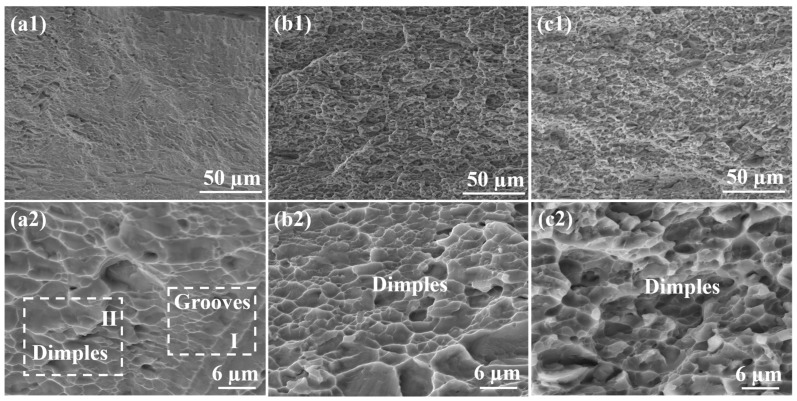
The fracture surfaces of HPT samples with 0.5 turn (**a1**,**a2**), 1 turn (**b1**,**b2**), and 3 turns (**c1**,**c2**).

Figure 8 displays the tensile fracture morphologies of HPT samples, which exhibit significant differences from those of the as-cast alloy, except for the HPT-processed sample through 0.5 turns, as presented in Figure 8(a1,a2). The fracture morphology also exhibited grooves and dimples, albeit shallower than those observed in the as-cast alloy, which corresponded with the previous research of Liu et al. [24]. As the shear strain increased, the dimples became smaller, as indicated in Figure 8(b1–c2), due to the refinement of the microstructures caused by the shear deformation, and the smooth fracture surface was observed. According to the earlier report, the shallower and smaller dimples implied a decrease in ductility [24], as displayed in Figure 6a. Meanwhile, it was believed that these small-sized dimples, resulting from an increased number of HPT turns, characterize intergranular fracture, consistent with previous reports [70,71,72]. Moreover, the fracture surface morphologies of the HPT samples through one turn and three turns consisted of a combination of fine and some coarse dimples, indicating a mixed fracture mode [73]. Therefore, the fracture mode of AlCoCrFeNi_2.1_ EHEAs remains a mixed fracture mode as the number of HPT turns increases.

## 4. Conclusions

The investigation into the impact of shear strain on the microstructure refinement and mechanical properties of AlCoCrFeNi_2.1_ EHEAs, subjected to HPT at room temperature under a pressure of 6 GPa through 0.5 to 3 turns, was conducted and compared with the characteristics of the initial material. The key conclusions drawn from this study are summarized as follows:

(1) Substantial microstructure refinement was achieved post-HPT, attributed to the generation of robust shear strain. XRD analysis delineated the refinement of the internal B2 phase via shear deformation, leading to the absence of B2 phase peaks.

(2) As the number of HPT turns increases, the shear strain due to HPT increases, resulting in a significant increase in the microhardness of the alloy. During the microhardness enhancement process, a two-stage saturation state emerged, denoting an equilibrium between defect generation and annihilation. Following three turns, the maximum saturation of microhardness reached approximately 575 HV, markedly surpassing that of the original sample.

(3) The UTS value of the as-cast AlCoCrFeNi_2.1_ EHEAs was approximately 1100 MPa. However, the internal microstructure of HPT specimens was refined, resulting in a UTS value of around 1900 MPa after three revolutions of HPT. Initially characterized by a mixed fracture mode comprising ductile and brittle fractures, which remained after HPT processing.

In a word, the HPT process leads to microstructure refinement by introducing shear deformation, which results in improved mechanical properties. However, when the shear deformation reaches a certain level, the refinement of microstructure and the improvement of mechanical properties reach a threshold. The HPT process offers a concept for the preparation of high-strength alloys and can be further integrated with heat treatment procedures to enhance plasticity, thereby catering to the demands for lighter weight materials in aerospace, automotive manufacturing, and various other industrial applications.

## Figures and Tables

**Table 1:** Chemical composition of the experimental EHEAs.

Element	Al	Co	Cr	Fe	Ni
at.%	16.39	16.39	16.39	16.39	34.43
wt.%	8.45	19.41	16.27	18.04	37.83

## Data Availability

The original contributions presented in the study are included in the article, further inquiries can be directed to the corresponding authors.
